# Supplementary material for: Genetic Mapping of Behavioral Traits Using the Collaborative Cross Resource
Source: Int J Mol Sci. 2022 Dec 30;24(1):682. doi: 10.3390/ijms24010682 (PMC9821145; doi:10.3390/ijms24010682)
Supplement: Supplementary file 1 [file ijms-24-00682-s001.zip › Supplementary Materials.pdf]

Supplementary Materials for

Genetic mapping of behavioral traits using the Collaborative Cross resource

Wei Xuan[1], Ling Zhang[1,*], Yu Zhang[1], Xiuping Sun[1], Jue Wang[1], Xianglei Li[1], Lingyan Zhang[1],

Xinpei Wang[1], Grant Morahan[1,2], Chuan Qin[1,3,*]

Correspondence:

zhangling@cnilas.org (Ling Zhang), qinchuan@pumc.edu.cn (Chuan Qin)

This PDF file includes:

Supplementary Figures S1 to S5

Supplementary Tables S1 to S11 (Please see attached Excel files)

Supplementary Figures and Legends

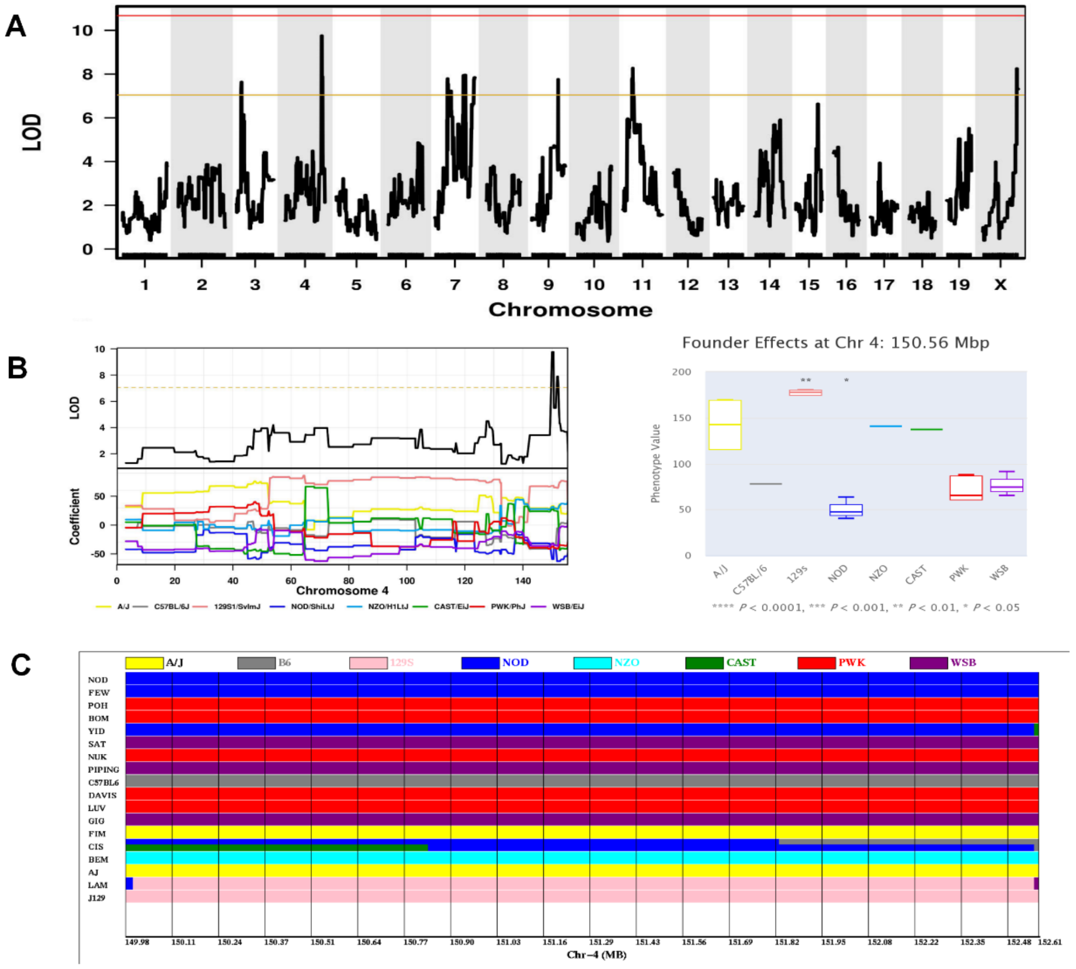

Supplementary Figure S1. Mapping an approaching significance QTL on Chr4 influencing the time spent immobile in an open field during a 5 min test period in the open field test. (A) Genome-wide LOD scores. The x-axis shows the chromosomal position and the y-axis shows the LOD scores. The solid red and orange horizontal lines indicate thresholds of 95% and 63% genome-wide significance, respectively. **(B)** LOD scores (upper left panel) on Chr4, and founder coefficient plot (bottom left panel) for Chr4. The dashed red horizontal line indicates thresholds of 95% significance. The association of founder haplotypes at 150.56 Mbp with this trait analyzed is shown in the right plot, indicating that the 129S1 haplotype significantly ($P < 0.01$) associated with the most time spend immobile of an open field during a 5 min test period, while NOD haplotype significantly ($P < 0.05$) associated with the least time. **(C)** Founder haplotypes in all CC strains at position 149.98–152.61 Mbp on Chr4. Strains are listed in the order from less time (top) to more time (bottom).

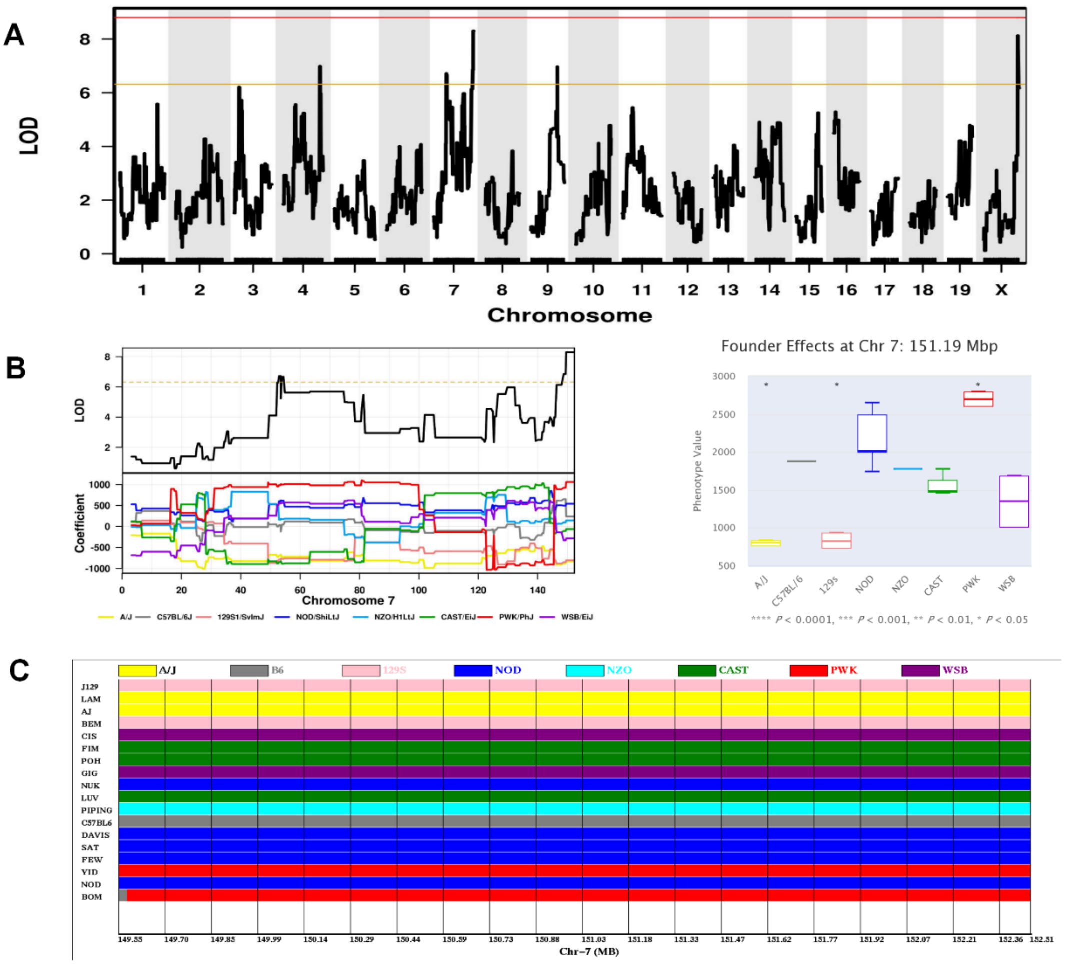

Supplementary Figure S2. Mapping an approaching significance QTL on Chr7 influencing the total distance traveled in the periphery of an open field during a 5 min test period in the open field test. **(A)** Genome-wide LOD scores. The x-axis shows the chromosomal position and the y-axis shows the LOD scores. The solid red and orange horizontal lines indicate thresholds of 95% and 63% genome-wide significance, respectively. **(B)** LOD scores (upper left panel) on Chr7, and founder coefficient plot (bottom left panel) for Chr7. The dashed red horizontal line indicates thresholds of 95% significance. The association of founder haplotypes at 151.19 Mbp with this trait analyzed is shown in the right plot, indicating that the 129S1 and A/J haplotype significantly ($P < 0.05$) associated with the shortest distance traveled in the periphery of an open field during a 5min test period, while PWK haplotype significantly ($P < 0.05$) associated with the longest distance. **(C)** Founder haplotypes in all CC strains at position 149.55–152.51 Mbp on Chr7. Strains are listed in the order from shorter distance (top) to longer distance (bottom).

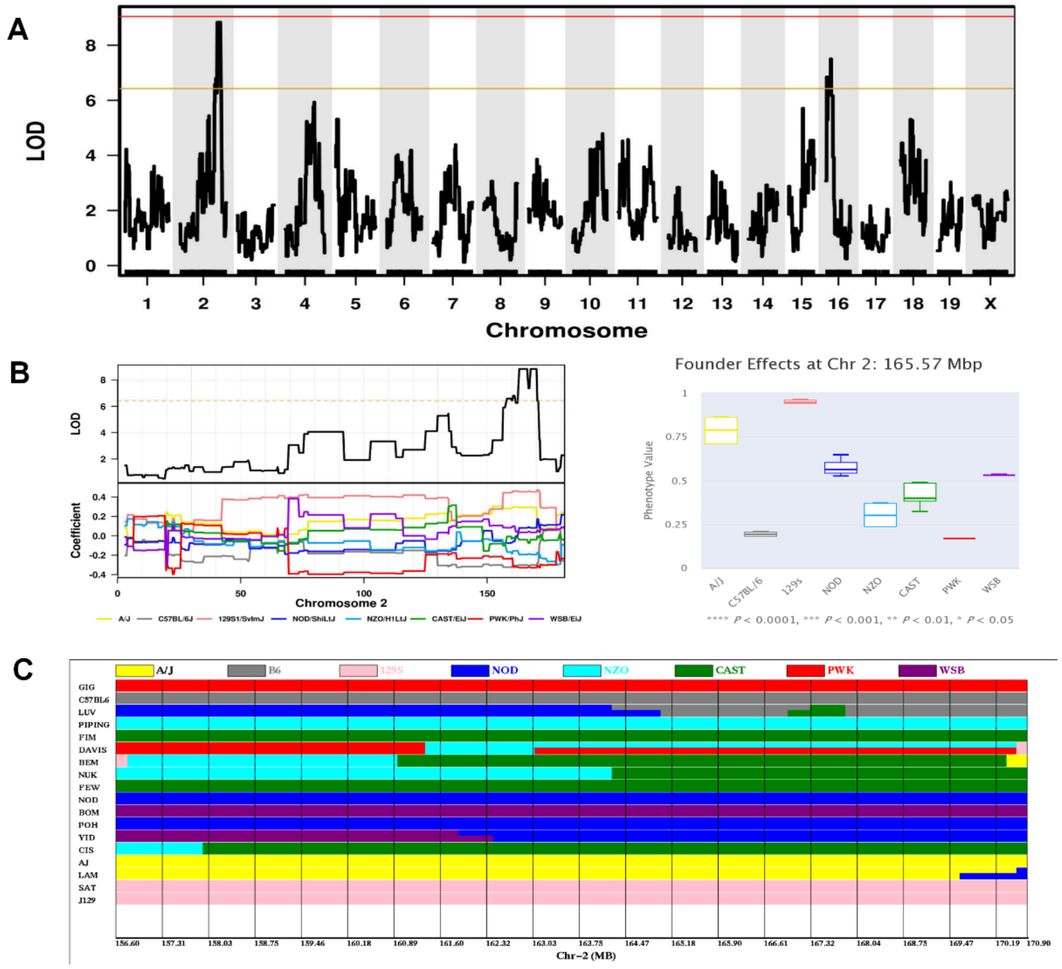

Supplementary Figure S3. Mapping an approaching significance QTL on Chr2 influencing the percentage of time spent freezing during the third post-shock interval on Day 4 for cued fear conditioning. (A) Genome-wide LOD scores. The x-axis shows the chromosomal position and the y-axis shows the LOD scores. The solid red and orange horizontal lines indicate thresholds of 95% and 63% genome-wide significance, respectively. **(B)** LOD scores (upper left panel) on Chr2, and founder coefficient plot (bottom left panel) for Chr2. The dashed red horizontal line indicates thresholds of 95% significance. The association of founder haplotypes at 165.57 Mbp with this trait analyzed is shown in the right plot, indicating that the 129 haplotype and A/J haplotype associated with the lowest percentage of time spent freezing. **(C)** Founder haplotypes in all CC strains at position 156.60–170.90 Mbp on Chr2. Strains are listed in the order from lower percentage (top) to higher percentage (bottom).

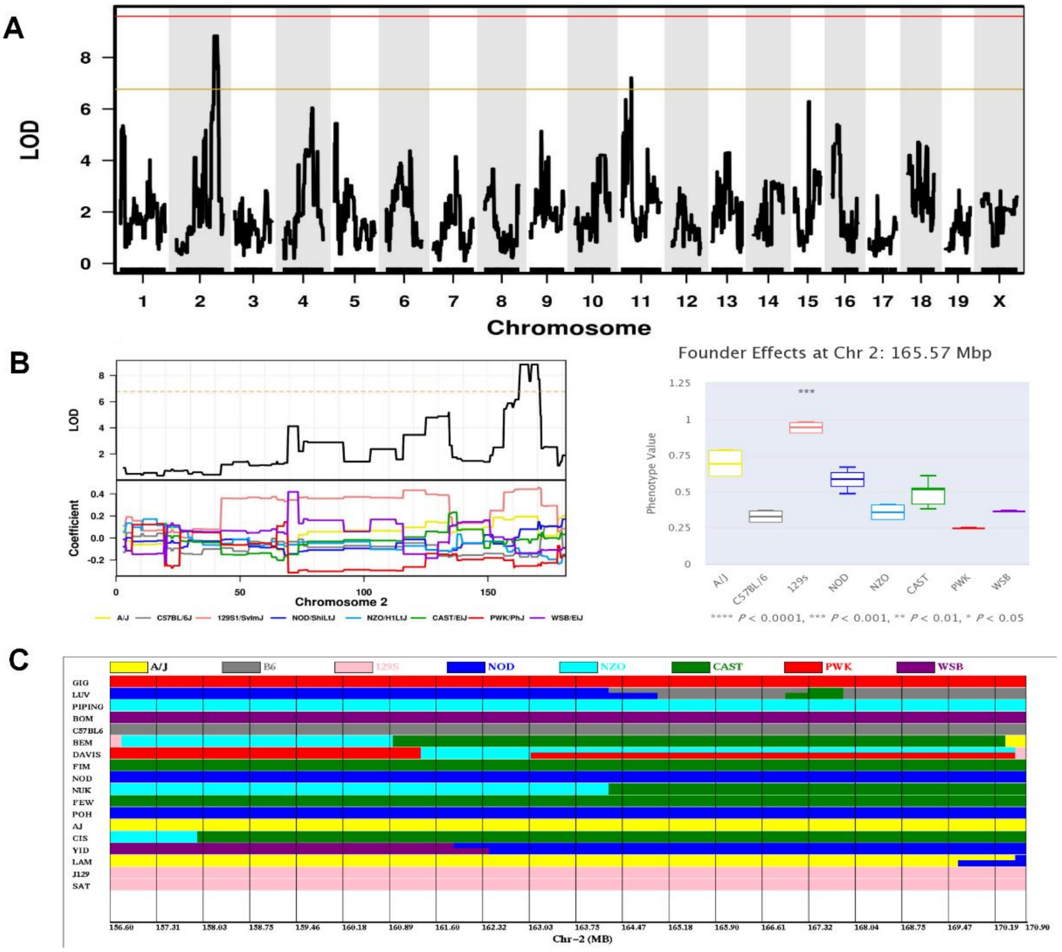

Supplementary Figure S4. Mapping an approaching significance QTL on Chr2 influencing the percentage of time spent freezing during the fifth post-shock interval on Day 4 for cued fear conditioning. (A) Genome-wide LOD scores. The x-axis shows the chromosomal position and the y-axis shows the LOD scores. The solid red and orange horizontal lines indicate thresholds of 95% and 63% genome-wide significance, respectively. **(B)** LOD scores (upper left panel) on Chr2, and founder coefficient plot (bottom left panel) for Chr2. The dashed red horizontal line indicates thresholds of 95% significance. The association of founder haplotypes at 165.57 Mbp with this trait analyzed is shown in the right plot, indicating that the 129 haplotype (being significant; P < 0.001) and A/J haplotype associated with the lowest percentage of time spent freezing. **(C)** Founder haplotypes in all CC strains at position 156.60–170.90 Mbp on Chr2. Strains are listed in the order from lower percentage (top) to higher percentage (bottom).

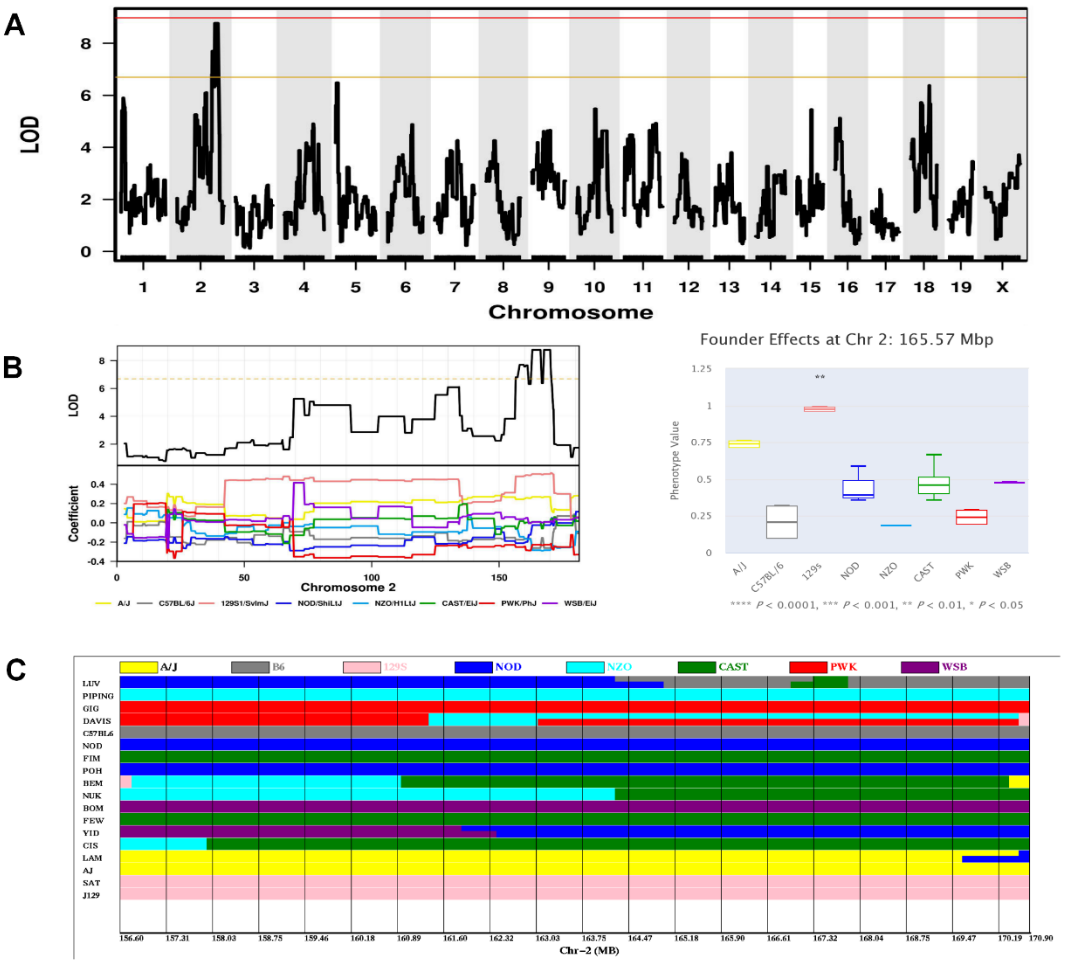

Supplementary Figure S5. Mapping an approaching significance QTL on Chr2 influencing the percentage of time spent freezing during five post-shock intervals on Day 4 for cued fear conditioning. (A) Genome-wide LOD scores. The x-axis shows the chromosomal position and the y-axis shows the LOD scores. The solid red and orange horizontal lines indicate thresholds of 95% and 63% genome-wide significance, respectively. **(B)** LOD scores (upper left panel) on Chr2, and founder coefficient plot (bottom left panel) for Chr2. The dashed red horizontal line indicates thresholds of 95% significance. The association of founder haplotypes at 165.57 Mbp with this trait analyzed is shown in the right plot, indicating that the 129 haplotype (being significant; $P < 0.01$) and A/J haplotype associated with the lowest percentage of time spent freezing. **(C)** Founder haplotypes in all CC strains at position 156.60–170.90 Mbp on Chr2. Strains are listed in the order from lower percentage (top) to higher percentage (bottom).
